# Conjugation of Therapeutic PSD-95 Inhibitors to the Cell-Penetrating Peptide Tat Affects Blood–Brain Barrier Adherence, Uptake, and Permeation

**DOI:** 10.3390/pharmaceutics12070661

**Published:** 2020-07-14

**Authors:** Mie Kristensen, Krzysztof Kucharz, Eduardo Felipe Alves Fernandes, Kristian Strømgaard, Morten Schallburg Nielsen, Hans Christian Cederberg Helms, Anders Bach, Malte Ulrikkaholm Tofte-Hansen, Blanca Irene Aldana Garcia, Martin Lauritzen, Birger Brodin

**Affiliations:** 1 Department of Pharmacy, University of Copenhagen, Universitetsparken 2, DK-2100 Copenhagen Ø, Denmark; hans.christian.helms@sund.ku.dk (H.C.C.H.); birger.brodin@sund.ku.dk (B.B.); 2 Department of Neuroscience and Pharmacology, University of Copenhagen, DK-2200 Copenhagen N, Denmark; kucharz@sund.ku.dk (K.K.); mlauritz@sund.ku.dk (M.L.); 3 Department of Drug Design and Pharmacology, University of Copenhagen, DK-2100 Copenhagen Ø, Denmark; eduardo.fernandes@sund.ku.dk (E.F.A.F.); kristian.stromgaard@sund.ku.dk (K.S.); anders.bach@sund.ku.dk (A.B.); mwc504@alumni.ku.dk (M.U.T.-H.); blanca.aldana@sund.ku.dk (B.I.A.G.); 4 Department of Biomedicine, Aarhus University, DK-8000 Aarhus C, Denmark; mn@biomed.au.dk

**Keywords:** brain peptide-drug delivery, blood–brain barrier, cell-penetrating peptide, Tat, stroke treatment

## Abstract

Novel stroke therapies are needed. Inhibition of the interaction between the postsynaptic density-95 (PSD-95)/disc large/ZO-1 (PDZ) domains of PSD-95 and the *N*-methyl-D-aspartate (NMDA) receptor has been suggested as a strategy for relieving neuronal damage. The peptides NR2B9c and *N*-dimer have been designed to hinder this interaction; they are conjugated to the cell-penetrating peptide Tat to facilitate blood–brain barrier (BBB) permeation and neuronal uptake. Tat-*N*-dimer exhibits 1000-fold better target affinity than Tat-NR2B9c, but the same magnitude of improvement is not observed in terms of therapeutic effect. Differences in BBB permeation by Tat-NR2B9c and Tat-*N*-dimer may explain this difference, but studies providing a direct comparison of Tat-NR2B9c and Tat-*N*-dimer are lacking. The aim of the present study was therefore to compare the BBB uptake and permeation of Tat-NR2B9c and Tat-*N*-dimer. The peptides were conjugated to the fluorophore TAMRA and their chemical stability assessed. Endothelial membrane association and cell uptake, and transendothelial permeation were estimated using co-cultures of primary bovine brain capillary endothelial cells and rat astrocytes. In vivo BBB permeation was demonstrated in mice using two-photon microscopy imaging. Tissue distribution was evaluated in mice demonstrating brain accumulation of TAMRA-Tat (0.4% ID/g), TAMRA-Tat-NR2B9c (0.3% ID/g), and TAMRA-Tat-*N*-dimer (0.25% ID/g). In conclusion, we demonstrate that attachment of NR2B9c or *N*-dimer to Tat affects both the chemical stability and the ability of the resulting construct to interact with and permeate the BBB.

## 1. Introduction

Ischemic stroke is a leading cause of death worldwide. Current therapy includes tissue plasminogen activator [1] and thrombectomy. However, these treatments do not stop the stroke-triggered excitotoxic reactions leading to cell death within the brain. Therefore, novel strategies to relieve neuronal damage, which potentially leads to long-term disabilities following ischemic stroke, are needed. During a stroke, interaction between postsynaptic density-95(PSD-95)/disc large/ZO-1 (PDZ) domains of PSD-95 and the *N*-methyl-D-aspartate (NMDA) receptor leads to nitric oxide production and neuronal death in the affected brain region [2]. Inhibition of this interaction may prevent neuronal damage after stroke. Aarts et al. and Cook et al. demonstrated that the 9-mer peptide corresponding to the C-terminus of the NMDA GluN2B subunit (NR2B9c) reduces cell death and improves cognition following experimental stroke in rats [3] and macaques [4], respectively. In addition, Bach et al. [5] developed the *N*-dimer, which has 1000-fold improved affinity for PDZ1-2 of PSD-95 compared to NR2B9c. To facilitate permeation across the blood–brain barrier (BBB) and internalization into neurons, NR2B9c and *N-*dimer are covalently conjugated to Tat [6], an 11-mer cationic cell-penetrating peptide (CPP) derived from the HIV transactivation of transcription protein that allows effective internalization into cells [7]. Schwarze et al. even suggested that Tat facilitates BBB permeation of the 120 kDa β-galactosidase protein [8].

A phase III clinical trial (ESCAPE NA-1) of Tat-NR2B9c (also named NA-1 or nerinetide) was completed in 2020 [9,10]. However, no neuroprotective effect was demonstrated in humans after administration of Tat-NR2B9c together with the trombolytic agent alteplase; possibly due to alteplase affecting the plasma concentration of Tat-NR2B9c. Tat-*N-*dimer (also named AVLX-144) entered Phase I clinical trials the same year. Compared to Tat-NR2B9c, Tat-*N*-dimer has been shown to further reduce the infarct volume and improve cognition upon administration to mice [5] and rats [11] subjected to experimental stroke. However, the 1000-fold improvement in target affinity of Tat-*N-*dimer in vitro compared to Tat-NR2B9c does not translate into a comparable magnitude in vivo [5]. Differences in the degree of BBB permeation could explain the differences between the in vitro and in vivo effects of Tat-NR2B9c and Tat-*N*-dimer. Tat-*N*-dimer delivery to the brain parenchyma has been verified quantitatively using fluorescent labels in mice [12] and rats [13]. The BBB permeation by fluorescently labelled Tat-*N*-dimer has also been qualitatively assessed in live mice by two-photon microscopy imaging [14]. However, studies directly comparing the BBB permeation potential of Tat-*N*-dimer and Tat-NR2B9c or investigating the underlying mechanism are lacking. In addition, studies addressing the effects of Tat on BBB permeation upon peptide cargo conjugation are limited.

**Figure 1:**
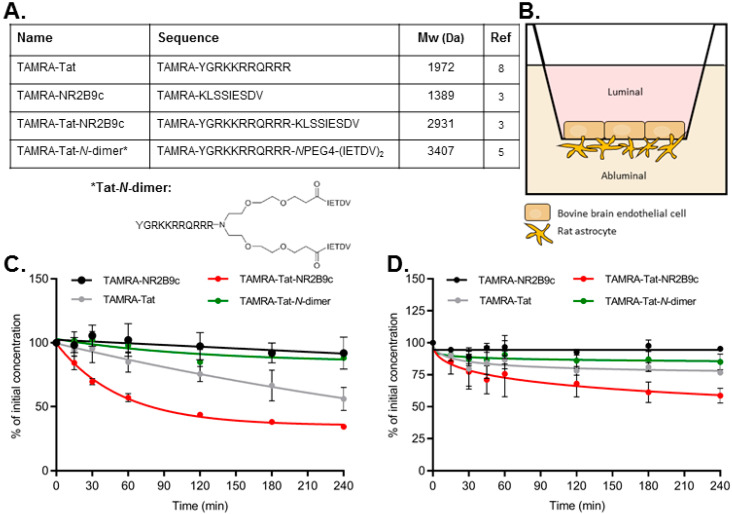
(**A**) Peptide constructs labelled with the fluorophore TAMRA and the Tat-*N*-dimer structure. (**B**) Illustration of the in vitro blood–brain barrier (BBB) model composed of primary bovine brain capillary endothelial cells in co-culture with primary rat astrocytes. (**C**) The peptide stability was evaluated during 4 h of incubation in 37 °C cell media supplemented with 10% fetal bovine serum (FBS) (N = 2) or (**D**) during luminal incubation on bovine brain endothelial cells co-cultured with rat astrocytes (as in (B)) in cell media without FBS (N = 3). Data are presented as mean ± SD.

NR2B9c and Tat-*N*-dimer are both positively charged at physiological pH but differ in structure. NR2B9c is a linear 9-mer peptide, whereas the *N*-dimer is composed of two 5-mer peptides conjugated to the N-terminus by a short custom-made nitrogen-containing polyethylene glycol (PEG) linker (Figure 1A). We hypothesized that attaching a peptide cargo to Tat will affect the BBB permeation of the resulting construct and that the nature of the cargo will affect the mode of BBB interaction and permeation. Monolayers of bovine brain capillary endothelial cells (BCECs) co-cultured with primary rat astrocytes were used to evaluate the barrier-interacting and permeating potential of fluorophore-labelled NR2B9c, Tat, Tat-NR2B9c, and Tat-*N*-dimer, as well the underlying mechanism. Two-photon microscopy was used to study the BBB permeation of the peptides in live mice. The tissue distribution was also evaluated in mice to quantify the level of peptide accumulation in the brain and off-target tissues.

## 2. Materials and Methods

TAMRA-NR2B9c (KLSSIESDV), TAMRA-Tat (YGRKKRRQRRR), and TAMRA-Tat-NR2B9c (YGRKKRRQRRRKLSSIESDV) were custom-made by Biomatik (Cambridge ON, Canada). TAMRA-Tat-*N*-dimer (YGRKKRRQRRR*N*PEG4(IETDV)_2_) was synthesized as described previously [5]. All peptides were prepared as HCl salts at >95% purity. Chemicals were obtained from Merck Life Science (Søborg, Denmark) unless otherwise stated.

### 2.1. Isolation of Rat Astrocytes

Astrocytes were isolated from 8–12, 2–3-day-old rats as described previously [15]. Briefly, cortices were isolated and gently passed through an 80-µm nylon mesh filter (Merck Millipore, Søborg, Denmark) into GIBCO Neural Cell Medium (Thermo Fischer, Bedford, MA, USA) supplemented with 2.5 mM L-glutamine, 6 mM D-glucose, 100,000 IU penicillin, and 26.2 mM NaHCO_3_ adjusted to pH 7.0 (Ast-DMEM) with 20% fetal bovine serum (FBS) (GE Healthcare, Brøndbyvester, Denmark) and triturated using a 13 G × 5″ steel cannula. The volume of the cell suspension was adjusted with Ast-DMEM containing 20% FBS and incubated in T25 flasks in a humidified incubator (37 °C, 5% CO_2_) for 3 weeks with the media changed every second day. During weeks 1, 2, and 3, the astrocytes were incubated in Ast-DMEM containing 20%, 15%, and 10% FBS, respectively. During week 3, the incubation medium was collected and stored at −20 °C as astrocyte conditioned media (ACM). At the end of week 3, the astrocytes were detached from the flasks using trypsin-ethylenediaminetetraacetic acid (EDTA) and spun down by centrifugation in Ast-DMEM containing 10% FBS (500× *g* for 5 min at room temperature). The astrocytes were re-suspended in FBS with 10% dimethyl sulfoxide (DMSO) and frozen at −80 °C overnight. After 24 h, the frozen vials were transferred to liquid nitrogen for long-term storage.

### 2.2. Bovine Brain Capillary Endothelial Cell and Rat Astrocyte Co-Culture

Isolation of bovine brain capillaries: capillaries from calves (<12 months of age) were isolated as described previously [16]. Briefly, the meninges were removed, and the grey matter scraped off the cortices using a razor blade and homogenized in ice-cold Dulbecco’s Modified Eagle Medium (DMEM) using a 40-mL Dounce tissue grinder set (Merck, St. Louis, MO, USA). Capillaries were isolated by passing the homogenate through a 160-µm nylon mesh filter (Merck Millipore, Darmstadt, Germany) and washed off the filters by flushing with DMEM complete medium (DMEM supplemented with 10% FBS, 1% non-essential amino acids, 100 U/mL penicillin/100 µg/mL streptomycin). The capillary fractions were centrifuged twice at 500× *g* for 5 min at room temperature. The pellet was re-suspended in digest mix (90 U/mL trypsin TRL, 200 U/mL collagenase type III, 170 U/mL DNAse I (Worthington Biochemical Corporation, Lakewood, NJ, USA) in DMEM complete) and incubated in a 37 °C water bath for 45 min. Digested capillaries were passed through a 200-µm nylon mesh filter (Merrem & la Porte, Zaltbommel, The Netherlands) prior to centrifugation at 500× *g* for 5 min at room temperature. Residual white matter was removed from the pellet by suction. The pellet was re-suspended in FBS with 10% DMSO and frozen at −80 °C overnight before storage in liquid nitrogen.

BCECs co-cultured with rat astrocytes: a batch of capillary suspension was thawed and the DMSO removed by centrifugation in DMEM complete at 500× *g* for 5 min at room temperature. The pellet was re-suspended in DMEM complete and transferred to a culture flask (T75) coated with collagen and fibronectin (both 0.01 mg/mL in PBS). The capillary fragments were allowed to settle for 4–6 h in a humidified incubator (37 °C, 10% CO_2_) before changing the media to growth medium(+) (DMEM complete/ACM (1:1) with 125 µg/mL heparin) supplemented with 4 µg/mL pyromycin. On day 3, the BCECs received fresh growth medium(+) and rat astrocytes were seeded on the bottom of collagen and fibronectin coated (both 0.1 mg/mL in PBS) Transwell^®^ plate permeable supports (Corning, Corning, NY, USA; 120,000 cells/1.12 cm^2^ support with 0.4 µm pore size) in DMEM complete (37 °C, 10% CO_2_). On day 5, the media in the Transwell^®^ plate was replaced by growth medium(-) (DMEM complete with 125 µg/mL heparin) and the endothelial cells detached from the flask using trypsin-EDTA (Thermo Fischer Scientific, Bedford, MA, USA). The remaining trypsin-EDTA was removed by centrifugation in DMEM complete at 1070× *g* for 5 min at room temperature. The endothelial cells were re-suspended in growth medium(-) and seeded on the apical side of the Transwell^®^ plate permeable supports (100,000 cells/support). On day 8, the medium was changed to differentiation medium (DMEM containing 50 mM *N-*Tris-(hydroxymethyl)methyl-2-aminoethanesulfonic acid (TES), 10% FBS, 1% non-essential amino acids, 100 µg/mL penicillin/streptomycin (100 U/mL), 2 mM L-glutamine, 312.5 µM 8-(4-CPT)cyclic adenosine monophosphate, 0.5 µM dexamethasone, and 17.5 µM RO-20-1724). On day 11, the bovine BBB model was ready for use as evident by the measured transendothelial electrical resistance (TEER) values for each filter prior to experimental start.

### 2.3. Mouse Brain Capillary Endothelial Cell and Rat Astrocyte Co-Culture

Isolation of mouse brain capillaries: four-week-old C57Bl/6 mice were sacrificed by cervical dislocation before being dipped in ethanol and the heads removed using scissors. The cerebral cortex was isolated and the meninges removed, followed by homogenization in ice-cold DMEM/F12 with penicillin and streptomycin using a 40-mL Dounce tissue grinder set (Merck, St. Louis, MO, USA). The homogenate was mixed with ice-cold 32% dextran in DMEM/F12 with 1:1 penicillin and streptomycin and kept on ice for 2 min before centrifugation at 2400× *g* at 4 °C for 20 min. Myelin and tissue pieces settled in the top layers were removed. The remaining fraction containing capillaries was re-suspended in DMEM containing 10% FBS and centrifuged at 800× *g* and 4 °C for 10 min to settle the remaining tissue. Capillaries left in the supernatant were re-suspended in DMEM containing 10% FBS and isolated by passing the suspension through a 40-µm nylon mesh filter and subsequently flushed off the filters with DMEM containing 10% FBS prior to centrifugation at 500× *g* for 5 min at room temperature. The pellet was re-suspended in digest mix (90 U/mL trypsin TRL, 200 U/mL collagenase type III, 170 U/mL DNAse I in DMEM containing 10% FBS) and incubated in a 37 °C water bath for 40 min. The volume of the digested capillary suspension was doubled with DMEM containing 10% FBS and centrifuged at 500× *g* for 5 min at room temperature. Capillaries were re-suspended in DMEM containing 10% FBS, transferred to a collagen and fibronectin (both 0.01 mg/mL in PBS) coated T25 flask, and placed in a humidified incubator (37 °C, 5% CO_2_).

Generation of mouse BCEC monolayers in co-culture with rat astrocytes: the capillary fragments were allowed to settle for 2–3 h before changing the media to DMEM containing 10% FBS and pyromycin (4 µg/mL). On day 3, the media was changed to DMEM containing 10% FBS and rat astrocytes seeded on the bottom of collagen and fibronectin coated (both 0.01 mg/mL in PBS) Transwell^®^ plate permeable supports (120,000 cells/1.12 cm^2^ support with 0.4 µm pore size). On day 5, the media in the Transwell^®^ plate was changed to Complete Mouse Endothelial Cell Medium (PELOBiotech, Planegg/Martinsried, Germany). The endothelial cells were detached from the flask using trypsin-EDTA. The remaining trypsin-EDTA was removed by centrifugation in DMEM containing 10% FBS at 500× *g* for 5 min at room temperature. The endothelial cells were re-suspended in Complete Mouse Endothelial Cell Medium and seeded on the apical side of the Transwell^®^ plate permeable supports (100,000 cells/support). On day 8, the medium was changed to differentiation medium. On day 10, the mouse BBB model was ready for use as evident by the measured transendothelial electrical resistance (TEER) values for each filter prior experimental start.

### 2.4. Peptide Stability Studies

Stability in serum-containing cell media: TAMRA-NR2B9c, TAMRA-Tat, TAMRA-Tat-NR2B9c, and TAMRA-Tat-*N*-dimer were dissolved in a small volume of ultrapure water and incubated in differentiation medium at a final concentration of 100 µM at 37 °C for 4 h. Samples (20 µL) were withdrawn at 0, 15, 30, 60, 120, 180, and 240 min. At room temperature, 50 µL of 6 M urea was added for 10 min to denature plasma proteins. To precipitate plasma proteins, 50 µL of 20% (*w/v*) trichloroacetic acid in acetone was added and the sample incubated at −20 °C for 10 min. The samples were spun down at 10,000× *g* for 5 min at 5 °C. The acetone phase containing the TAMRA-peptides was filtered (pore size 0.22 µm) prior to analysis by UPLC using a Waters Acquity system with a C18 BEH reverse phase column (BEH C18 column, 2.1 mm × 50 mm × 1.7 µm). Mobile phase A consisted of 95% ultrapure water and 5% acetonitrile (ACN) with 0.1% trifluoroacetic acid (TFA) and mobile phase B of 95% ACN and 5% ultrapure water with 0.1% TFA. A linear gradient was used (min/%B): 0/5%, 12/45%, 12.1/100%, 13/100%, 13.1/5%, 15/5%, with a flow rate of 0.45 mL/min over 15 min with UV detection at 214 and 498 nm. Lower limits of detection are 6.2 µM for TAMRA-NR2B9c, 0.8 µM for TAMRA-Tat, 1.6 µM for TAMRA-Tat-NR2B9c, and 1.6 µM for TAMRA-Tat-*N*-dimer.

Stability on the in vitro bovine BBB model: the differentiation medium was gently aspirated from the BCEC monolayers and replaced with 300 and 600 µL of 37 °C FBS-free differentiation medium containing 50 µM peptide on the luminal and abluminal side, respectively. The cells were incubated at 37 °C with horizontal shaking (90 rpm) for 4 h. Samples (10 µL) were withdrawn at 0, 10, 20, 30, 45, 60, 120, 180, and 240 min and two volumes of ice-cold ACN were immediately added prior to analysis by HPLC using an AdvanceBio Peptide Map column (C18, 4.6 × 150 mm, 2.7 µm particle size; Agilent, Glostrup, Denmark) connected to a Prominence system (LC-20 AD, SIL20 AC, SPD-M20A PDA, and CTO-20A modules; Shimadzu, Kyoto, Japan). Mobile phase A consisted of 95% ultrapure water and 5% ACN with 0.1% TFA and mobile phase B of 95% ACN and 5% ultrapure water with 0.1% TFA. A linear gradient of 20–80% B with a flow rate of 20 mL/min was applied over 8 min with detection at 220 and 543 nm.

### 2.5. In Vitro Transport and Cellular Uptake

Transport across bovine or mouse BCEC monolayers co-cultured with rat astrocytes was carried out over 3 h at 37 °C with horizontal shaking (90 rpm). TEER was measured at room temperature using an Endohm 12 cup (World Precision Instruments, Sarasota, FL, USA) connected to a Millicell-ERS device (Millipore, Bedford, MA, USA). TEER was measured before application of the peptides and after each experiment. The bovine model had initial TEER values ranging from 771 to 1332 Ω·cm^2^, whereas the mouse model had initial TEER values ranging from 136 to 217 Ω·cm^2^. The monolayers and experimental solutions were equilibrated to 37 °C and the experiment initiated by spiking the media in the apical compartment with 2 mM TAMRA-peptide stocks alone or together with 10 mg/mL FITC-labelled dextran (3–5 kDa) stock to achieve final test concentrations of 100 µM TAMRA-NR2B9c, TAMRA-Tat, TAMRA-Tat-NR2B9c, or TAMRA-Tat-*N*-dimer and 1 mg/mL FITC-dextran.

Transport and cell uptake of FITC-dextran (bovine model): after 3 h of incubation with FITC dextran alone or together with TAMRA-Tat, TAMRA-Tat-NR2B9c, or TAMRA-Tat-*N*-dimer, 100 µL samples were withdrawn from the basolateral compartment into a black clear-bottom 96-well plate. FITC fluorescence was measured using a NovoStar fluorescence plate reader (BMG Labtech, Offenburg, Germany) with excitation/emission set to 494/518 nm. Transported FITC-dextran was calculated using a standard curve (10–160 nM) that was freshly prepared in differentiation medium supplemented with 10% FBS. To visualize the cell uptake of FITC-dextran, the filters were washed thrice with Hanks Balanced Salt Solution (HBSS) supplemented with 10 mM 4-(2-hydroxyethyl)-1-piperazineethanesulfonic acid (HEPES) and 0.05% bovine serum albumin (BSA) and adjusted to pH 7.4 (hHBSS) prior to 15 min incubation with 3% paraformaldehyde in phosphate buffered saline (PBS) at room temperature with horizontal shaking (90 rpm) for fixation. The filters were washed with PBS thrice for 5 min, mounted on glass slides under coverslips using Immu-Mount™ mounting media (Thermo Fischer Scientific, Bedford, MA, USA), and imaged using single wavelength excitation on a Leica SP2 laser-scanning confocal microscope (Leica Microsystems, Wetzlar, Germany).

Transport, membrane adherence, and cell uptake of TAMRA-NR2B9c, TAMRA-Tat, TAMRA-Tat-NR2B9c, and TAMRA-Tat-*N*-dimer (bovine model): after 3 h of incubation with the individual peptides, 100 µL samples from the abluminal compartment were transferred to a Corning^®^ black transparent bottom 96-well plate. TAMRA fluorescence was measured using a NovoStar fluorescence plate reader (BMG Labtech, Offenburg, Germany) with excitation/emission set to 542/568 nm. The transported peptide was calculated using standard curves ranging from 31 nM to 2 µM. All peptide standard curves were subjected to 3 h of incubation at 37 °C with horizontal shaking (90 rpm) in differentiation media withdrawn from BCECs co-cultured with rat astrocytes.

Two filters from each BCEC batch were subjected to quantification of the TAMRA-peptide adhering to the BCEC plasma membrane and peptide taken up by the BCECs and astrocytes. The remaining filter from each batch was applied to immunocytochemistry for qualitative visualization of cell uptake of TAMRA-NR2B9c, TAMRA-Tat, TAMRA-Tat-NR2B9c, and TAMRA-Tat*-N-*dimer. The peptide solutions were removed from the cells and the filters washed thrice with 37 °C hHBSS. To isolate the peptide fraction adhering to the plasma membrane, the BCECs were placed on ice and incubated with 300 µL ice-cold acetic acid buffer (15 µM sodium acetate trihydrate, 10 µM glucose, 1 µM ethylenediaminetetraacetic acid (EDTA), 1.3 µM MgSO_4_ heptahydrate, 5 µM KCl, 100 µM NaCl, 100 µM acetic acid, 1% BSA, pH 3) for 5 min. The acetic acid buffer solution was gently pipetted up and down twice prior to collection of 100 µL samples into a black clear-bottom 96-well plate for analysis. The remaining acetic acid buffer solution was removed from the luminal chamber and the cells washed thrice with ice-cold hHBSS. To isolate the peptide fraction being taken up by the BCECs and astrocytes, the filter inserts were cut out and 150 µL of ice-cold lysis buffer (10 mM Tris-HCl, 0.25 M sucrose, 1 mM EDTA, 1 mM ethylene-bis(oxyethylenenitrilo)tetraacetic acid tetrasodium (EGTA), 2% Tergitol^™^, Complete^®^ inhibitor tablet) added prior to incubation for 10 min on ice. The cells were homogenized using a pellet pestle (Merck, St. Louis) and subjected to centrifugation at 10,000× *g* at 4 °C for 10 min. The 100 µL supernatant was transferred into a black clear-bottom 96-well plate. TAMRA fluorescence was measured using a NovoStar fluorescence plate reader (BMG Labtech, Offenburg, Germany) with excitation/emission set to 542/568 nm. The molar amount of membrane-adhered peptide and peptide taken up by the cells was calculated using standard curves freshly prepared in acetic acid buffer and lysis buffer, respectively, in concentrations ranging from 40 nM to 2.5 µM.

Transport and cellular accumulation of TAMRA-NR2B9c, TAMRA-Tat, TAMRA-Tat-NR2B9c, and TAMRA-Tat-*N*-dimer (mouse model): a 100 µL aliquot of the experimental solution was sampled from the abluminal compartment at 15, 30, 60, 120, and 180 min and transferred to a black clear-bottom 96-well plate. The withdrawn volume was replaced with media.

Transported peptide was calculated based on fluorescence using the same method as described for peptides applied to the bovine model. Subsequently, the peptide solutions were removed from the cells and the filters washed thrice with 4 °C hHBSS. To isolate the peptides accumulated in the cell fraction, the filter inserts were cut out and 250 µL of ice-cold lysis buffer added. The peptides were retrieved and quantified as described for the cell uptake fraction obtained following the permeation study using the bovine model.

### 2.6. Immunocytochemistry

To visualize the TAMRA-peptide taken up by the endothelial cells and astrocytes, the remaining filters (one per batch) from the permeation study using the in vitro bovine BBB model were prepared for confocal microscopy. After removal of the peptide solutions, the cells were fixed with 3% paraformaldehyde in PBS for 15 min at room temperature with horizontal shaking (90 rpm). The cells were washed thrice with PBS prior to permeabilization in 0.1% Triton X-100 in PBS for 5 min and blocking in 2% BSA in PBS for 30 min at room temperature with horizontal shaking (90 rpm). The filter inserts were cut out, divided into smaller pieces, and transferred to a 48-well plate to stain zonula ocludens-1 (ZO-1) in the BCECs and F-actin in the astrocytes. Filter pieces with BCECs facing upwards were incubated with a primary antibody directed against ZO-1 (1:100 in PBS with 2% BSA; Thermo Fischer Scientific, Bedford, MA, USA, no. 617300) for 2 h at room temperature. Subsequently, the filters were washed thrice for 5 min in PBS with 2% BSA prior to 30 min incubation with an Alexa-488-conjugated secondary antibody (1:100 in PBS with 2% BSA; Thermo Fischer Scientific, Bedford, MA, USA, no. A11008). Filter pieces with astrocytes facing upwards were incubated with Alexa-488-conjugated phalloidin (1:133 in PBS with 2% BSA; Thermo Fischer Scientific, Bedford, MA, USA) for 30 min at room temperature and washed thrice for 5 min in PBS with 2% BSA. The filter pieces were mounted on glass slides under coverslips using Immu-Mount™ mounting media and imaged using single wavelength excitation a Zeiss 510 laser-scanning confocal microscope (Carl Zeiss, Jena, Germany).

### 2.7. Cellular Viability

The cellular viability of filter-grown BCECs co-cultured with rat astrocytes was evaluated following 3-h incubation with TAMRA-NR2B9c, TAMRA-Tat, TAMRA-Tat-NR2B9c, and TAMRA-Tat-*N*-dimer using an XTT assay as directed by the manufacturer. Briefly, the XTT labelling mixture was prepared by mixing electron coupling reagent with XTT labelling reagent at a 1:50 ratio. After peptide incubation, the monolayers were washed twice with 37 °C hHBSS. We applied 300 µL and 1 mL 37 °C hHBSS to the apical and basolateral compartments, respectively, prior to the addition of 150 µL XTT labelling mixture. The filters were incubated at 37 °C with horizontal shaking (90 rpm) for 1 h. Two 100 µL samples were withdrawn from the apical compartments and the absorbance measured at 466 nm using a SPECTROstar Nano plate reader (BMG Labtech, Offenburg, Germany). The relative cell viability was calculated as follows:

$$\mathrm{Relative}\;\mathrm{viability}\;\left(\%\right)=\frac{A_{sample}-A_{SDS}}{A_{buffer}-A_{SDS}}\times 100\%$$

where *A_sample_* is the absorbance of the test sample, *A_SDS_* is the absorbance of the negative control (100% dead cells), and *A_buffer_* is the absorbance of the positive control (100% viable cells).

### 2.8. Live Cell Peptide Uptake

BCECs were obtained from a batch of isolated capillary fragments and cultured as described in Section 2.2. Following trypsinization on day 5, the BCECs were re-suspended in differentiation medium and 90,000/cm^2^ seeded on collagen- and fibronectin-coated (both 10 µg/mL in PBS) LabTek^®^ microscope slides (Merck, Søborg, Denmark). The cells were left in the incubator (10% CO_2_, 37 °C) for 48 h prior to use. On the day of the experiment, the cell medium was spiked with 2 mM stocks of TAMRA-peptides in order to achieve a final test concentration of 100 µM for TAMRA-NR2B9c and 50 µM for TAMRA-Tat, TAMRA-Tat-NR2B9c, and TAMRA-Tat-*N*-dimer prior to 60 min incubation at 37 °C with horizontal shaking (90 rpm). The test solutions were removed and pre-warmed media containing 300 nM Hoechst and Lysotracker^®^ (1:20,000; Thermo Fischer Scientific, Bedford, MA, USA) applied to the cells for 10 min at 37 °C. The cells were gently washed twice in 37 °C hHBSS and immediately imaged in FluoBrite medium (Thermo Fischer Scientific, Bedford, MA, USA). Images were captured using an Olympus IX-83 fluorescent microscope (Olympus, Hamburg, Germany) equipped with a Yokogawa CSU-X spinning disc unit (Yokogawa, Tokyo, Japan) and Andor iXon Ultra 897 camera (Oxford Instruments, Abingdon, Great Britain) using cellSens software (Olympus, Hamburg, Germany).

### 2.9. Uptake in Mouse Cortical Neurons

Isolation and culture of primary cortical neurons: cortical neuronal cultures were prepared as described by Hertz et al. [15]. Pregnant NMRI mice (Envigo, Huntingdon, United Kingdom) were euthanized by cervical dislocation and cortices isolated from 15-day-old fetuses under aseptic conditions. The tissue was subjected to trypsinization (0.2 mg/mL in PBS) at 37 °C for 10 min. Trypsinization was inactivated by trituration of the tissue in a DNase solution (75 IU/mL) containing soybean trypsin inhibitor (0.52 mg/mL). The cells were suspended in DMEM supplemented with 24.5 mM KCl, 12 mM glucose, 7.3 mM *p*-aminobenzoic acid, and 10% FBS (In Vitro, Fredensborg, Denmark) prior to seeding (600,000 cells/cm^2^) onto poly-D-lysine-coated cover slips (Corning, Corning, NY, USA) placed in Petri dishes (1.5 mL/22 mm; NUNC, Roskilde, Denmark). Cultures were kept at 37 °C and 5% CO_2_. To prevent astrocyte proliferation [17], 20 µM cytosine arabinoside was added after 24 h. Glucose was supplemented twice during the culture period, and a minimum glucose concentration of 12 mM was maintained. The cortical neurons were used for experiments after 7 days in culture.

Peptide uptake into primary cortical neurons: the medium was spiked with 2 mM stocks of TAMRA-Tat, TAMRA-Tat-NR2B9c, or TAMRA-Tat-*N*-dimer to final concentrations of 1 and 5 µM. The plates were gently circulated horizontally in order to distribute the peptides within the media prior to 1 h of incubation at 37 °C and 5% CO_2_. The peptide solutions were removed and the cells gently washed once with 37 °C hHBSS. The cells were fixated with 3% paraformaldehyde in PBS for 15 min, permeabilized with 0.1% Triton X-100 in PBS for 5 min, and blocked in 2% BSA in PBS for 30 min at room temperature with horizontal shaking (90 rpm). Subsequently, the filters were washed thrice for 5 min in PBS with 2% BSA, incubated with Alexa-488-conjugated phalloidin (1:133 in PBS with 2% BSA) for 30 min, and washed thrice for 5 min in PBS with 2% BSA. The cover slips were mounted on glass slides with Immu-Mount™ mounting media and the neurons imaged using a Leica SP2 laser-scanning confocal microscope (Leica Microsystems, Wetzlar, Germany).

### 2.10. In Vivo Two-Photon Imaging

Animals and operational procedure: We used 12–14-week-old C57BL/6 mice (24–26 g) and performed the surgical procedures as described previously [18]. Anesthesia was induced by intraperitoneal (i.p.) injection of xylazine (10 mg/kg body weight; ScanVet, Fredensborg, Denmark) and ketamine (60 mg/kg body weight; MSD Animal Health, København, Denmark), and maintained by supplementary i.p. injections of ketamine (30 mg/kg body weight) every 20–25 min. The trachea was surgically exposed, and a custom-made metal tube inserted into the trachea lumen for mechanical respiration (180–220 μL volume; 190–240 strokes/min) with oxygen-supplemented air (1.5–2 mL/min) using a MiniVent Type 845 ventilator (Harvard Apparatus, Holliston, MA, USA). Catheters were inserted into the femoral vein and the femoral artery. The incisions were closed with surgical staples. After cannulation, the animal was placed in a prone position. The scalp was removed and FeCl_3_ (0.62 M) administered to clean off the periosteum. The exposed skull was subsequently washed with saline, dried, and attached to a custom-made metal plate using a cyanoacrylate gel (Henkel’s LOCTITE^®^, Düsseldorf, Germany). The animal head was mounted onto the imaging stage insert and craniotomy performed using a dental drill (7000 rpm; 4 mm lateral and 1 mm posterior to bregma, Ø = 3 mm). The bone flap and dura were carefully removed, and the surface of the brain gently washed with artificial cerebrospinal fluid (120 mM NaCl, 2.8 mM KCl, 1 mM Na_2_HPO_4_, 0.876 mM MgCl_2_, 22 mM NaHCO_3_, 1.45 mM CaCl_2_, 2.55 mM glucose, pH 7.4, 37 °C, 5% CO_2_). We applied 1% type III-A agarose to the brain surface, and a Menzel glass coverslip (4 × 4 mm, 0.08-mm thick; Thermo Fischer Scientific, Bedford, MA, USA) was positioned to seal the major part of the craniotomy, leaving only an opening for the glass microelectrode used in the electrophysiological assessment. The mouse was transferred to the imaging stage. Following the insertion of the glass microelectrode, the anesthesia was switched to α-chloralose administered intravenously at a constant flow of 50 mg/kg body weight/hour. The mouse was left for 25 min for anesthesia equilibration before imaging. During all procedures, the animal was maintained at 37 °C using a YSI-451rectal thermistor probe (E-Z Systems, Palmer, PA, USA) with a TC-1000 Mouse Proportional controlling unit connected to a heating pad (08-13013; CWE Incorporated, Palmer, PA, USA). To ensure optimal animal physiology and level of anesthesia, end-tidal respiratory CO_2_ was measured using a Capnograph Type 340 (Harvard Apparatus, Holliston, MA, USA), and mean arterial blood pressure was monitored using a BP-1 pressure monitor (World Precision Instruments, Sarasota, FL, USA).

Imaging of BBB permeation: TAMRA-Tat, TAMRA-Tat-NR2B9c, and TAMRA-Tat-*N*-dimer were administered as 60 µL bolus injections via the catheter inserted into the femoral artery (3 nmol/g body weight). Following 1 h of peptide circulation, 50 µL of 0.5% 2 MDa FITC-dextran was administered as a control for mechanical damage. The imaging was performed using an SP5 upright laser scanning microscope equipped with a 20 × 1.0 N.A. water-immersion objective (Leica Microsystems, Wetzlar, Germany). Images were obtained in a hyperstack mode, i.e., in a series of Z-stacks in 1-min intervals. Each Z-stack underwent a reduction of dimensionality (maximum signal intensity projection along the Z-axis). TAMRA and FITC were excited with a Millennia Pro MaiTai HP Ti:Sapphire two-photon laser (Spectra Physics, Santa Clara, CA, USA) tuned to 870 nm. The signal was acquired by two multi-alkali NDD photomultipliers after 525–560 and 560–625 nm band pass filters for FITC-dx and TAMRA fluorescence, respectively. Data were exported from LAS AF software (v. 4.4, Leica Microsystems, Wetzlar, Germany) to ImageJ v. 1.48u4 (NIH). To assess the pharmacokinetics of the peptides, the fluorescence over time was measured from small regions of interest (ROIs) placed on the brain parenchyma in areas devoid of vasculature (n_parenchyma_ = 80 ROIs/animal) and on the blood vessels (n_bloodvessel_ = 10 ROIs/animal). The signal was collected for each Tat compound and for the subsequent BBB integrity control with 2 MDa FITC-dextran. The traces from each animal were averaged and the area under the curve (AUC) calculated per animal for peptide- and FITC-dextran accumulation, as well as clearance from the bloodstream.

Brain electrophysiology*:* An aCSF-filled Micropipette Puller Model P-97 single-barreled filament borosilicate glass electrode with Ag/AgCl wire (electrode resistance, 1.8–2.0 MΩ; Sutter Instrument, Navato, CA, USA) was inserted into the cerebral cortex ≈50 μm below the brain surface. The reference electrode was placed into the neck muscle posterior to the craniotomy. The signal was sampled at a frequency of 20 kHz using Spike2 software (v. 7.02a; CED). The electrocorticographic activity was obtained after 10× amplification and 0–3000 Hz low-pass filter using an AP311 analogue amplifier (Warner Instruments, Hamden, CT, USA), with subsequent additional 100× amplification and 0.5 Hz high-pass filter using an AC/DC NL 106 amplifier equipped with an NL 125/126 filter (Harvard Bioscience, Holliston, MA, USA). The baseline was the average from the 5 min signal prior to the administration of peptides. The end-point was the average of the 5 min signal collected 1 h post-injection.

### 2.11. Tissue Distribution

TAMRA-Tat, TAMRA-Tat-NR2B9c, and TAMRA-Tat-*N*-dimer were dissolved in 0.9% NaCl and administered as a 100 µL bolus injection (3 nmol/g body weight) into the tail vein of 12-week-old male NMRI mice (27–33 g). Following 1 h of circulation, the mice were deeply anesthetized by i.p. injection of 0.05 mL/10 g body weight Ketaminol/Dexdormitor (2.25 mg ketamine/0.03 mg meteoidin per 10 g body weight in 0.9% NaCl). The chest cage was opened and the mice transcardially perfused with 0.01 M PBS (pH 7.4) prior to isolation of the brain, heart, lungs, spleen, kidney, liver, and 2 cm of the upper part of the intestine. All tissues were immediately put on ice and kept at −80 °C until analysis.

The tissues were homogenized in 1 mL of ice-cold lysis buffer using a 7-mL Dounce tissue grinder set (Merck, St. Louis, MO, USA). Homogenates were left on ice for 10 min prior to centrifugation at 14,000× *g* and 4 °C for 10 min. Supernatants (100 µL) were transferred to a black clear-bottom 96-well plate for quantification of the TAMRA-peptides using a NovoStar fluorescence plate reader (BMG Labtech, Offenburg, Germany) with excitation and emission set to 542 and 568 nm, respectively. The molar peptide amounts were calculated using standard curves that were freshly prepared in lysates from relevant blank tissue at concentrations ranging from 30 nM to 2 µM.

### 2.12. Data and Statistical Analysis

Data processing and statistical analysis were carried out using Microsoft Office Excel and GraphPad Prism version 7 (GraphPad software, San Diego, CA, USA), respectively. The Student’s paired *t*-test, one-way analysis of variance (ANOVA) with Dunnett post-test, or two-way ANOVA with Sidak post-test was applied. A *p*-value < 0.05 was considered significant. Data are presented with standard deviation (SD) or standard error of the mean (SEM) with N representing the number of replicates and *n* representing the number of experiments.

### 2.13. Ethical Information

Bovine brains for the isolation of endothelial cells were obtained as a by-product from a Danish slaughterhouse (Mogens Nielsen Kreaturslagteri A/S, Herlufmagle, Denmark) following Danish legislation (BEK no. 135 of 2104-02-14; European legislation identifier/eli/lta/2014/135). Rats used for the isolation of astrocytes were euthanized according to Danish legislation on animal experimentation (BEK no. 12 of 2106-01-07; European legislation identifier/eli/lta/2016/12). Two-photon imaging in live mice was performed according to Danish Animal Research License no. 2019-15-0201-01655. Experimental procedures for tissue distribution studies in mice were conducted according to Danish Animal Research License no. 2014-15-0201-00031. All animal procedures were designed to comply with the ARRIVE guidelines.

## 3. Results

### 3.1. Chemical Stability Differs among the Peptide Constructs

Tat, NR2B9c, and Tat-NR2B9c are linear amino acid sequences, whereas the Tat-*N*-dimer is more complex and composed of the Tat sequence and two pentapeptides connected by a PEG linker (Figure 1A). NR2B9c was included in parts of this study to investigate whether the cellular uptake of Tat-conjugates was due to a unique ability of Tat to permeate the plasma membrane. All peptide constructs were N-terminally labelled with the fluorophore TAMRA for imaging and quantification. An in vitro model composed of primary bovine BCECs co-cultured with primary rat astrocytes (Figure 1A) was used to study the chemical stability of the constructs upon contact with the BBB.

**Figure 2:**
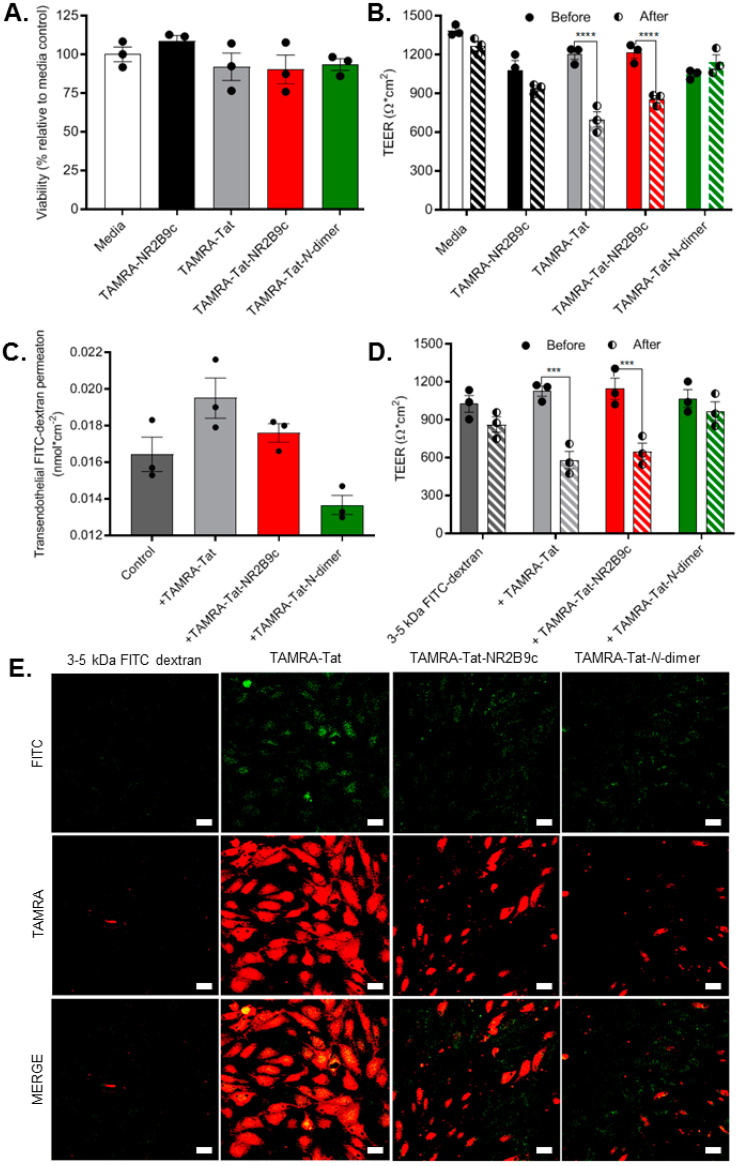
(**A**) The cellular viability was assessed following application of 100 µM TAMRA-NR2B9c, TAMRA-Tat, TAMRA-Tat-NR2B9c, or TAMRA-Tat-*N*-dimer to the in vitro BBB model for 3 h. (**B**) The barrier integrity was evaluated by measuring transendothelial electrical resistance (TEER) before and after application of the peptides (100 µM). (**C**) Transport of 3–5 kDa FITC-dextran was quantified after 3 h of incubation on the in vitro BBB model alone (1 mg/mL; control) or in the presence of the peptide (100 µM). (**D**) The barrier integrity was assessed by measuring TEER before and after application of 4 kDa FITC-labelled dextran (1 mg/mL) together with the peptide (100 µM). Data are presented as mean ± SEM (N = 3, *n* = 3). *** *p* < 0.001 and **** *p* < 0.0001 (one-way ANOVA with Dunnett’s multiple comparisons test). (**E**) Representative confocal microscopy images of 4 kDa FITC-dextran uptake into the endothelial cells after 3 h of incubation and cell fixation. Maximal z-stack projections, scale bar: 20 µm.

First, the ability of the constructs to resist degradation by enzymes present in serum was assessed. TAMRA-NR2B9c and TAMRA-Tat-*N*-dimer exhibited a low degree of degradation with 92.0% ± 12.6% and 88.1% ± 1.8% of the initial concentration, respectively, remaining after 4 h of incubation in serum-supplemented cell media (Figure 1C and Figure S1A,D). TAMRA-Tat and TAMRA-Tat-NR2B9c appeared to be more prone to degradation with 56.0% ± 8.9% and 34.3% ± 1.7% remaining, respectively, following the 4 h incubation period (Supplementary Materials Figure S1B,C). One of the degradation products appeared to correspond with the TAMRA moiety (Figure S1E). BCECs express γ-glutamyltranspeptidase (γ-GT) and alkaline phosphatase (ALP) [19]. The bovine BCEC/rat astrocyte co-culture BBB model has also demonstrated ALP activity [20]. The stability of the peptides was evaluated using the in vitro BBB model with luminal incubation in cell media without FBS (Figure 1D and Figure 2A). A similar pattern was observed after 4 h of luminal incubation on the BCECs (Figure 1D) compared to incubation in the serum-containing media (Figure 1C). TAMRA-Tat-NR2B9c was the least stable protein (58.6% ± 13.1% remaining), followed by TAMRA-Tat (76.9% ± 5.6% remaining), TAMRA-Tat-*N*-dimer (85.0% ± 6.0% remaining), and TAMRA-NR2B9c (95.7% ± 1.6% remaining). All peptides remained stable over the 4 h incubation period in PBS alone (Figure S2).

### 3.2. TAMRA-Tat and TAMRA-Tat-NR2B9c Affect Barrier Integrity but Not the Permeability of 3–5 kDa Dextrans across the Brain Endothelial Monolayer In Vitro

Effects on cell viability (Figure 2A) and barrier integrity (Figure 2B) were assessed after 3 h of incubation with TAMRA-NR2B9c, TAMRA-Tat, TAMRA-Tat-NR2B9c, and TAMRA-Tat-*N*-dimer on the primary bovine BCECs co-cultured with primary rat astrocytes (Figure 1B). The cell viability was assessed by measuring the mitochondrial activity using a commercially available assay. The barrier integrity was monitored by recording the transendothelial electrical resistance (TEER) before and after the transport study and the assessment of 3–5 kDa dextran permeability. Incubation with TAMRA-NR2B9c did not decrease cell viability. A slight, but non-significant, decrease in cell viability was observed upon incubation with TAMRA-Tat (*p* = 0.320), TAMRA-Tat-NR2B9c (*p* = 0.114), or TAMRA-Tat-*N-*dimer (*p* = 0.382) compared to the media-treated control cells (Figure 2A). Similarly, incubation with TAMRA-NR2B9c or TAMRA-Tat-*N*-dimer did not affect the barrier integrity, whereas incubation with TAMRA-Tat or TAMRA-Tat-NR2B9c resulted in a significant decrease in TEER (Figure 2B).

To evaluate whether the observed decrease in TEER leads to paracellular permeation by the Tat-conjugates, 3–5 kDa FITC-labelled dextrans were applied to the luminal chamber with TAMRA-Tat, TAMRA-Tat-NR2B9c, or TAMRA-Tat-*N*-dimer in the in vitro BBB model (Figure 1B). The amount of FITC-dextran in the abluminal chamber after 3 h of incubation was quantified (Figure 2C). A slight increase in FITC-dextran permeation was observed upon application with TAMRA-Tat or TAMRA-Tat-NR2B9c (*p* = 0.067 and *p* = 0.631, respectively) compared to the application of FITC-dextran alone (Figure 2C). The transendothelial permeation by FITC-dextran did not increase upon co-administration with TAMRA-Tat-*N*-dimer. In parallel, effects on the barrier integrity resulting from application of FITC-dextran alone or together with the Tat-conjugates was evaluated by measuring the TEER before and after the permeation study (Figure 2D). Application of FITC-dextran alone or with TAMRA-Tat-*N-*dimer did not affect barrier integrity. The effects on barrier integrity upon co-application of FITC-dextran with TAMRA-Tat and TARMA-Tat-NR2B9c were similar to the effects without FITC-dextran (Figure 2B).

To evaluate whether the Tat-conjugated peptides affected the cell uptake of FITC-dextran, the BCECs were fixated and inspected for FITC fluorescence by confocal microscopy after the permeation study (Figure 2E). A low level of FITC-dextran uptake was observed in the in vitro BBB model following application of FITC-dextran alone. A markedly higher uptake was observed when the FITC dextran was applied in the presence of the Tat-conjugates. The FITC fluorescence in the endothelial cells appeared in discrete regions of the cytosol, indicating that the conjugates promoted vesicular uptake of the FITC-labelled dextrans. TAMRA-Tat appeared to promote FITC-dextran uptake to a greater extent than TAMRA-Tat-NR2B9c and TAMRA-Tat-*N*-dimer.

### 3.3. Cargo-Conjugation to Tat Affects Blood–Brain Barrier Adherence, Uptake, and Permeation

**Figure 3:**
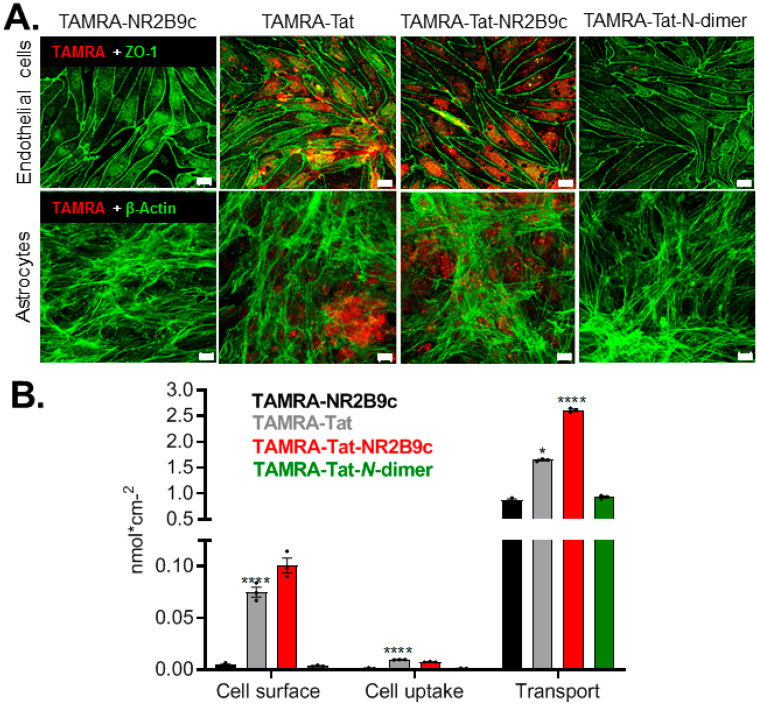
Adherence, uptake, and permeation following application of 100 µM TAMRA-NR2B9c, TAMRA-Tat, TAMRA-Tat-NR2B9c, or TAMRA-Tat-*N*-dimer to the in vitro BBB model for 3 h. (**A**) Following cell fixation, peptide uptake into the endothelial cells and astrocytes was inspected via TAMRA fluorescence using confocal microscopy with co-staining of ZO-1 and β-actin, respectively. Maximal z-stack projections, scale bar: 10 µm. (**B**) The total peptide adhering to the cell surface, taken up by the cells, and transported across the barrier was quantified. Data are presented as mean ± SEM (N = 2–3, *n* = 3). * *p* < 0.05, **** *p* < 0.0001 compared to TAMRA-NR2B9c (one-way ANOVA with Dunnett’s multiple comparisons test for TAMRA-Tat-NR2B9c and TAMRA-Tat-*N*-dimer, and paired *t*-test for TAMRA-Tat).

The ability of TAMRA-NR2B9c, TAMRA-Tat, TAMRA-Tat-NR2B9c, and TAMRA-Tat-*N*-dimer to internalize into BCECs and to permeate brain endothelial cell monolayers was evaluated using BCECs co-cultured with astrocytes (Figure 1B). The endothelial cells and astrocytes were fixated after 3 h of incubation with the peptides and subsequently inspected for TAMRA fluorescence using confocal microscopy (Figure 3A). Staining of ZO-1, a tight junction-associated protein, and the cytoskeleton (β-actin) was included for morphological inspection of the endothelial cells and astrocytes, respectively. TAMRA-NR2B9c uptake was not observed in the endothelial cells or the astrocytes. Uptake of TAMRA-Tat and TAMRA-Tat-NR2B9c was observed in both cell types, whereas a low level of TAMRA-Tat-*N*-dimer uptake was observed in the endothelial cells, but not in the astrocytes. Uptake of the Tat-conjugated peptides was evaluated after just 15 min of incubation, demonstrating uptake of TAMRA-Tat and TAMRA-Tat-NR2B9c in both the endothelial cells and astrocytes (Figure S3). Minor uptake of TAMRA-Tat-*N*-dimer was observed in the endothelial cells, but not in the astrocytes, following 15 min of incubation. TAMRA as a single entity was not internalized into the endothelial cells or the astrocytes (Figure S4).

Next, the fraction of peptide adhering to the cell surface, the fraction being taken up by the endothelial cells or astrocytes, and the fraction being transported across the barrier was quantified based on TAMRA fluorescence (Figure 3B). Minute amounts of TAMRA-NR2B9c and TAMRA-Tat-*N*-dimer were detected in fractions containing cell surface-adhered peptide and peptide taken up by the cells. Significantly larger amounts of TAMRA-Tat and TAMRA-Tat-NR2B9c appeared to both adhere to the cell surface and internalize into the cells compared to TAMRA-NR2B9c and TAMRA-Tat-*N*-dimer. TAMRA-Tat and TAMRA-Tat-NR2B9c permeated the barrier model to a greater extent than TAMRA-NR2B9c upon assessment via fluorescence. No significant difference in the barrier-permeating properties was observed when comparing TAMRA-Tat-*N*-dimer and TAMRA-NR2B9c (*p* = 0.798) in the abluminal content. A similar tendency was observed when applying the peptides to an in vitro BBB model based on primary mouse BCECs in co-culture with primary rat astrocytes (Figure S5). TAMRA-Tat-NR2B9c permeated the mouse BBB model to a greater extent than TAMRA-NR2B9c following 3 h of incubation, whereas the transendothelial permeation by TAMRA-Tat was similar and TAMRA-Tat-*N*-dimer lower than that of TAMRA-NR2B9c (Figure S5A). Both TAMRA-Tat and TAMRA-Tat-NR2B9c accumulated in the cell fraction of the mouse BBB model to a greater extent than TAMRA-NR2B9c (Figure S5B), which is similar to the observations in the bovine BBB model (Figure 3B).

In order to reach their biological target, Tat-NR2B9c and Tat-*N*-dimer must permeate the brain capillary endothelium and, subsequently, the plasma membrane of the neurons within the brain parenchyma. Therefore, the ability of Tat to facilitate the uptake of NR2B9c and *N*-dimer into primary cortical neurons was investigated. The peptides were applied to the neuronal cultures at low micromolar concentration (1 µM) due to expected limited BBB permeation (Figure 3B). Neuronal uptake was observed for all of the Tat-conjugated peptides following 1 h of incubation (Figure S6).

### 3.4. Tat and Tat-Cargo Constructs Are Taken up by BCECs in Vesicular Structures

**Figure 4:**
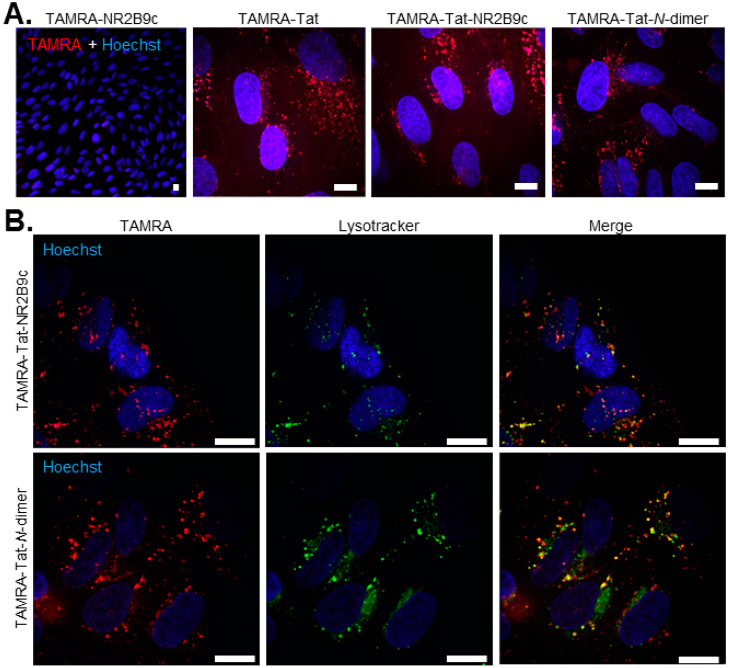
(**A**) Live cell imaging to visualize peptide uptake into bovine brain capillary endothelial cells (BCECs) following the application of 100 µM TAMRA-NR2B9c, TAMRA-Tat, TAMRA-Tat-NR2B9c, or TAMRA-Tat-*N*-dimer for 1 h at 37 °C. (**B**) Potential segregation of TAMRA-Tat-NR2B9c and TAMRA-Tat-*N*-dimer into lysosomes was studied by inclusion of LysoTracker. Single xy sections, scale bar: 20 µm.

The overall mechanism, by which the Tat-conjugated peptides enter endothelial cells, whether via direct membrane translocation or vesicular uptake, was studied in monocultures of primary bovine BCECs (Figure 4). Peptide uptake was imaged using live cells due to the earlier reported effect of fixation on the cellular distribution of CPPs [21]. Therefore, no conclusions should be drawn on the mechanism leading to peptide uptake following the permeation study in which the cells are subjected to fixation prior to imaging (Figure 3A). LysoTracker™ was included to investigate whether the cell-internalized TAMRA-Tat-NR2B9c and TAMRA-Tat-*N*-dimer segregated to lysosomes for degradation (Figure 4B). No uptake of TAMRA-NR2B9c was observed after 1 h of incubation. TAMRA-Tat, TAMRA-Tat-NR2B9c, and TAMRA-Tat-*N*-dimer were all taken up into vesicular structures to a similar extent (Figure 4A), which contrasts the uptake levels observed in the co-culture BBB model (Figure 2E and Figure 3). Inclusion of LysoTracker™ resulted in some co-staining with TAMRA-Tat-NR2B9c and TAMRA-Tat-*N*-dimer (Figure 4B), revealing segregation to lysosomes.

### 3.5. Cargo Conjugation to Tat Affects the Blood–Brain Barrier Permeation in Live Mice

**Figure 5:**
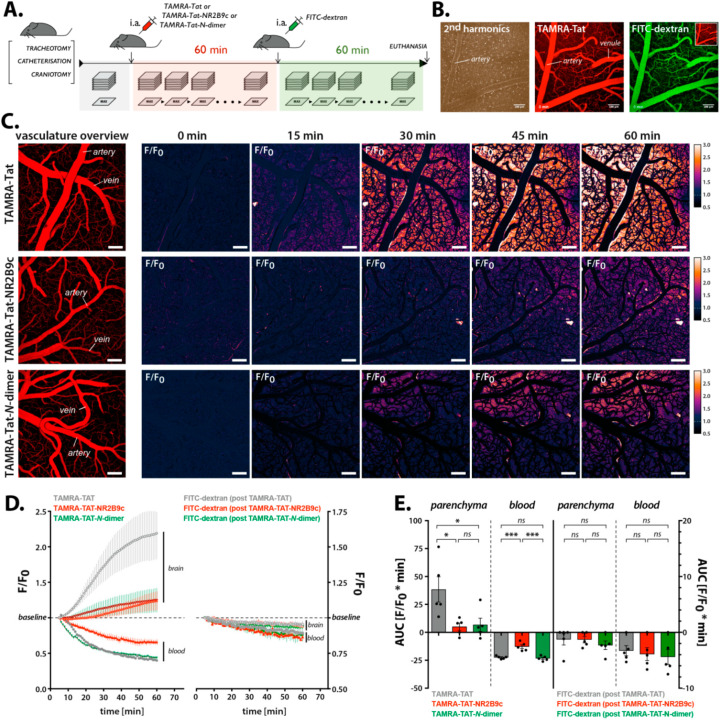
BBB permeation by the TAMRA-Tat-conjugated peptides was studied in C57BL/6 mice using two-photon fluorescence imaging. (**A**) Schematic timeline of the experiment, including surgical procedures and peptide administration, followed by administration of 2 MDa FITC-dextran as a control for mechanical damage of the BBB. (**B**) Second harmonics generation (**left**), baseline TAMRA-Tat fluorescence (**middle**), and baseline FITC-dextran fluorescence (**right**). (**C**) Time-lapse recordings of relative increases in fluorescence in the brain parenchyma following administration of TAMRA-Tat (**top**), TAMRA-Tat-NR2B9c (**middle**), or TAMRA-Tat-*N*-dimer (3 nmol peptide/g body weight). Scale bar: 100 µm. (**D**) Kinetic profiles presented as changes in fluorescence of the TAMRA-Tat-conjugated peptides (**left**) and FITC-dextran (**right**) in the brain parenchyma and vessels over time. (**E**) Total change in fluorescence of the TAMRA-Tat-conjugated peptides (**left**) and FITC-dextran (**right**) in the brain parenchyma and blood vessels presented as the area under the curve (AUC) obtained from the kinetic profiles. Data are presented as mean ± SEM (N = 5). * *p* < 0.05, *** *p* < 0.001 (two-tailed unpaired *t*-test).

The uptake of TAMRA-Tat, TAMRA-Tat-NR2B9c, and TAMRA-Tat-*N*-dimer into the living brain was estimated using two-photon fluorescence imaging in anesthetized C57BL/6 mice (Figure 5A–E). The mice were administered 3 nmol peptide/g body weight via the femoral artery. FITC-dextran (2 MDa) was administered after 1 h of peptide circulation in order to exclude mechanical damage causing peptide permeation of the BBB (Figure 5A). A second harmonics generation signal was initially recorded to ensure the presence of pial arteries and veins in the imaging area (Figure 5B). In addition, baseline TAMRA and FITC fluorescence was recorded within the imaging area.

Each of the Tat-conjugated peptides induced a gradual increase in TAMRA fluorescence in the brain parenchyma (Figure 5C) with the kinetic profiles outlined in Figure 5D. TAMRA-Tat exhibited significantly better BBB permeation (32.21 ± 10.17 F/F_0_ * min) compared to TAMRA-Tat-NR2B9c (5.54 ± 2.95 F/F_0_ * min) and TAMRA-Tat-*N-*dimer (6.90 ± 3.90 F/F_0_ * min; Figure 5E). No difference was observed in the fluorescence accumulation in the parenchyma originating from TAMRA-Tat-NR2B9c and TAMRA-Tat-*N-*dimer (*p* = 0.788). The clearance of TAMRA-Tat-NR2B9c as determined by decreased fluorescence in the blood stream (−4.97 ± 1.26 F/F_0_ min) was significantly lower than that of TAMRA-Tat (−17.85 ± 1.71 F/F_0_ min) and TAMRA-Tat-*N-*dimer (−15.02 ± 0.55 F/F_0_ min; Figure 5E). The clearance profiles of TAMRA-Tat and TAMRA-Tat-*N-*dimer did not differ (*p* = 0.153). The FITC-dextran administered following 1 h of peptide circulation did not permeate the BBB (Figure 5D,E). The electrocorticogram of exhaled CO_2_ and mean arterial blood pressure were monitored in order to follow the physiological condition of the animals when subjected to peptide circulation. No dramatic effects on the physiological parameters were observed after 1 h of peptide circulation (Figure S7).

### 3.6. Tat-Conjugated Peptides Exhibited Broad Tissue Distribution in Mice

**Figure 6:**
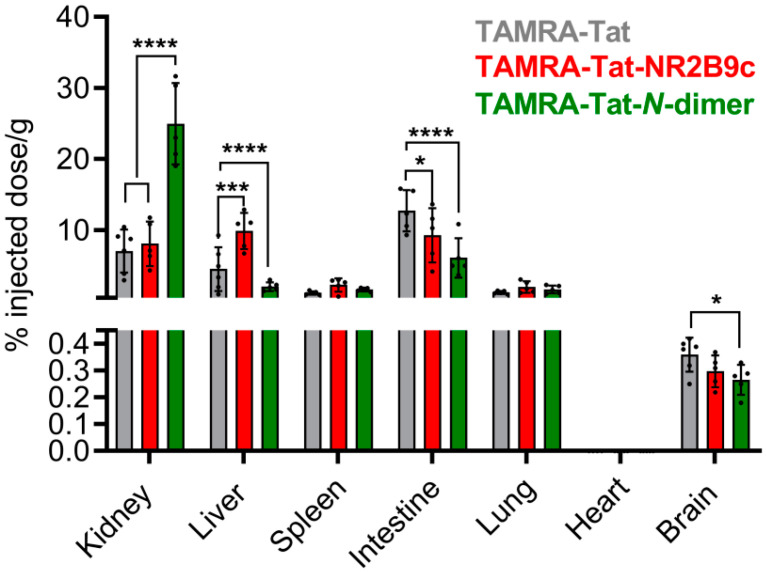
Biodistribution of TAMRA-Tat, TAMRA-Tat-NR2B9c, and TAMRA-Tat-*N*-dimer in tissues isolated from NMRI mice following tail vein administration of 3 nmol peptide/g body weight and 1 h of circulation with quantification via TAMRA fluorescence. Data are presented as mean ± SD (N = 4–6). * *p* < 0.05, *** *p* < 0.001, **** *p* < 0.0001 (two-way ANOVA with Tukey’s multiple comparisons test for kidney, liver, spleen, intestine, lung, and heart; unpaired *t*-test for brain).

The total brain accumulation of the Tat-conjugated peptides and accumulation in off-target tissues was assessed 1 h after tail vein administration of 3 nmol peptide/g body weight to awake NMRI mice (Figure 6). Tissue accumulation of TAMRA-Tat, TAMRA-Tat-NR2B9c, and TAMRA-Tat-*N*-dimer was quantified based on TAMRA fluorescence in whole tissue homogenates. Therefore, brain peptide accumulation represents peptide adherence to the capillaries, peptide entrapment within the endothelial cells, and peptide uptake into the brain parenchyma.

Administration of Tat-conjugates resulted in the accumulation of TAMRA fluorescence in the kidneys, liver, spleen, intestines, lungs, and brain, but not in the heart. The peptides primarily accumulated in the kidneys, liver, and intestines. Administration of TAMRA-Tat-*N*-dimer gave rise to more fluorescence in the kidney than TAMRA-Tat or TAMRA-Tat-NR2B9c. In the liver, TAMRA-Tat-NR2B9c appeared to accumulate to a greater extent than TAMRA-Tat and TAMRA-Tat-*N*-dimer, whereas TAMRA-Tat accumulation was superior in the intestines. The accumulation of fluorescence in the spleen and lungs did not differ among the Tat-conjugates. Administration of TAMRA-Tat gave rise to slightly higher levels of TAMRA fluorescence in the brain homogenates than the brains from mice administered TAMRA-Tat-NR2B9c (*p* = 0.131) or TAMRA-Tat-*N*-dimer (*p* = 0.030). All Tat-conjugates exhibited significant accumulation in the brain homogenates compared to blank brain tissue homogenates (unpaired *t*-test, *p* < 0.0001).

## 4. Discussion

The peptide-based drugs Tat-NR2B9c and Tat-*N*-dimer have demonstrated potential for relieving detrimental effects within the brain tissue following ischemic stroke. Tat-*N*-dimer has 1000-fold better target affinity in vitro than Tat-NR2B9c. The same large difference was not observed when examining the therapeutic effects of the two compounds in vivo, though Tat-*N*-dimer is still more potent than Tat-NR2B9c. This could be related to differences in their ability to permeate the BBB. Thus, the objective of this study was to investigate the ability of different peptide cargos attached to Tat to permeate the BBB in vitro and in vivo.

Peptide-based drugs are prone to chemical degradation when presented to enzymes during systemic circulation. Therefore, the stability of the peptide constructs was examined in serum-supplemented media and during incubation in a state-of-the-art in vitro BBB model made up of primary BCECs co-cultured with primary rat astrocytes. Despite their common peptide nature, the stability varied markedly. Almost no degradation of TAMRA-NR2B9c or TAMRA-Tat-*N*-dimer was observed, whereas TAMRA-Tat and TAMRA-Tat-NR2B9c degraded significantly. The half-life of ^125^I-labelled Tat (YGRKKRRQRRR) in mouse serum has been determined to be 2.7 min [22]. Another study demonstrated that ^111^In-DOTA-labelled Tat (GRKKRRQRRRPPQ) has a half-life of 528 min in human serum [23]. This indicates that the Tat-labelling strategy affects the resulting chemical stability in biological matrices. In the present study, we showed better stability when TAMRA-Tat was conjugated to the *N*-dimer. Moreover, we demonstrated that TAMRA-Tat-*N*-dimer exhibits better stability than TAMRA-Tat-NR2B9c, which correlates with earlier findings in 90% human plasma [5]. Therefore, the chemical half-life of Tat constructs can be improved by conjugation to certain molecules. Notably, Bach et al. reported half-lives of 1100 min and 4900 min for Tat-NR2B9c and Tat-*N-*dimer, respectively, in human serum. Again, the longer half-life reported for Tat-NR2B9c compared to TAMRA-labelled Tat-NR2B9c in the present study could be due to the N-terminal fluorophore conjugation.

We observed limited interaction with the plasma membrane and low endothelial uptake of TAMRA-Tat-*N*-dimer compared to TAMRA-Tat and TAMRA-Tat-NR2B9c. Macchi et al. demonstrated that Tat self-associates in solution [24], whereas Monreal et al. reported improved uptake into primary hippocampal neurons upon Tat dimerization compared to Tat as a single entity [25]. Thus, Tat conjugation to the *N-*dimer moiety, but not NR2B9c, may promote self-association into larger multimeric complexes, which ultimately shields the cationic Tat moiety, restricting interactions with the negatively charged plasma membrane constituents. Additional studies are required to verify these findings. Alternatively, interaction with plasma proteins may prevent TAMRA-Tat-*N*-dimer from interacting with the plasma membrane, which Kosuge et al. suggested explains the decreased uptake of poly-arginine into HeLa cells with increasing serum concentrations [26].

In the present study, TAMRA-Tat and TAMRA-Tat-NR2B9c appeared to permeate the in vitro BBB model to a greater extent than TAMRA-NR2B9c and TAMRA-Tat-*N*-dimer when assessed by TAMRA fluorescence. This observation is in line with the observed uptake of TAMRA-Tat and TAMRA-Tat-NR2B9c, but not TAMRA-NR2B9c and TAMRA-Tat-*N*-dimer, into the astrocytes. In contrast, in live mice, TAMRA-Tat permeated the BBB to a greater extent than TAMRA-Tat-NR2B9c, implying that cargo conjugation to Tat limits the BBB permeation by the resulting construct. In addition, TAMRA-Tat-*N*-dimer permeated the BBB to the same extent as TAMRA-Tat-NR2B9c in live mice, which was not the case in vitro. Notably, we documented extensive degradation of TAMRA-Tat and TAMRA-Tat-NR2B9c, but not TAMRA-Tat-*N*-dimer. Therefore, we questioned whether the fluorescence originates from intact TAMRA-Tat and TAMRA-Tat-NR2B9c or their TAMRA-containing metabolites, including TAMRA as a single entity. Application of TAMRA alone to the co-culture BBB model did not result in cell uptake of the fluorophore, which is in agreement with findings reported by Zhao et al., who showed that HeLa cells do not take up TAMRA as a single entity [27]. Thus, the observed peptide uptake into the endothelial cells and astrocytes reflects the uptake of either intact peptide or peptide-metabolites conjugated to TAMRA. However, we cannot completely exclude that part of the TAMRA fluorescence reflecting the in vitro or in vivo BBB permeation originates from paracellular permeation by TAMRA as a single entity. Birch et al. demonstrated previously that fluorophore conjugation to the CPP penetratin affects its cellular distribution and cell viability [28]. Hedegaard et al. documented an altered mode of membrane interaction upon fluorophore conjugation [29], whereas Walter et al. showed that radiolabeling impacts the ability of CPPs to traverse cell membranes [30]. Therefore, the label must be carefully considered during study design and the comparison of peptide uptake into cells based on different labels should be avoided. In the present study, TAMRA conjugation to peptides allowed us to visualize their cell uptake and to study the underlying mechanism, as well as BBB permeation in live mice. However, we cannot exclude that TAMRA may influence the cell uptake and/or BBB-permeating properties of NR2B9c, Tat, Tat-NR2B9c, and Tat-*N*-dimer.

Permeation of the BBB by drugs may occur via a transcellular route or through the paracellular space between endothelial cells. Cationic CPPs, such as Tat, may exploit adsorptive-mediated transcytosis, a commonly suggested route for transport of positively charged macromolecules across the BBB [31,32]. Tyagi et al. [33] and Poon et al. [34] suggested electrostatic interactions with negatively charged plasma membrane constituents as the initial trigger for vesicular uptake of CPPs into cells. In line with this suggestion, we demonstrated that the degree to which the Tat-conjugates adhered to the plasma membrane correlated with their ability to be taken up by the endothelial cells. A number of studies have demonstrated endocytic uptake of CPPs [35,36,37,38], including Lim et al., who documented endocytic uptake of Tat into primary splenocytes [39]. In line with these studies, we observed vesicular uptake of the Tat-conjugated constructs in live bovine BCECs. Moreover, we demonstrated that the presence of TAMRA-Tat, TAMRA-Tat-NR2B9c, and TAMRA-Tat-*N*-dimer boosted the cell uptake of 3–5 kDa FITC-dextran. Complexation between the peptides and FITC-dextran could, in principle, facilitate cell uptake of the latter, as exploited for intracellular delivery of oligonucleotides [40,41]. However, TAMRA-Tat-NR2B9c and TAMRA-Tat-*N*-dimer did not co-localize with FITC-dextran upon cell uptake, excluding that FITC-dextran uptake occurs only when complexed with the peptides. Instead, the Tat-conjugated peptides may affect the baseline level of vesicle formation at the plasma membrane in BCECs.

TAMRA-Tat-*N*-dimer appeared to be taken up by the primary BCECs cultured on microscope slides to the same extent as TAMRA-Tat and TAMRA-Tat-NR2B9c, which contradicts the observed degree of uptake in the in vitro BBB model. Recently, Toth et al. demonstrated that co-culture of BCECs with astrocytes affects the endo-lysosomal sorting system [42]. Whether the presence of the astrocytes affects the uptake of TAMRA-Tat-*N*-dimer in the co-culture BBB model, but not in the BCECs when cultured alone on microscope slides, requires further study before conclusions can be drawn. Johnsen et al. [43] and Gao et al. [44] showed previously that segregation to lysosomes within the capillary endothelial cells is an obstacle for successful macromolecular BBB permeation when exploiting receptor-mediated uptake. We demonstrated some segregation of TAMRA-Tat-NR2B9c and TAMRA-Tat-*N*-dimer to lysosomes. If the peptides are taken up by the BCECs via adsorptive-mediated endocytosis, a fraction may experience degradation in lysosomes, and the remaining may by exocytosed at the abluminal membrane.

Alternatively, drug BBB permeation may occur via diffusion through the paracellular space. Little attention has been given to this route when exploiting CPPs as vectors for drug delivery across biological barriers. Lindgren et al. demonstrated that the CPPs transportan and transportan 10 affect the monolayer integrity of the Caco-2 intestinal cell culture model [45]. Applying TAMRA-Tat and TAMRA-Tat-NR2B9c to the in vitro BBB model resulted not only in cellular uptake of the peptides, but also a decrease in barrier integrity. However, only a minor increase in FITC-dextran permeation was observed upon application with TAMRA-Tat and TAMRA-Tat-NR2B9c. Therefore, paracellular permeation may not be the main mechanism underlying the observed in vitro and in vivo BBB permeation by TAMRA-Tat, TAMRA-Tat-NR2B9c, and TAMRA-Tat*-N*-dimer.

Several studies have concluded that Tat exhibits broad tissue distribution after systemic circulation [8,22,23,46], which was confirmed in the present study. Limited information is available with respect to the tissue distribution of Tat upon conjugation to a therapeutic peptide moiety, such as NR2B9c and *N*-dimer. We showed that the specific cargo to which Tat was conjugated affected the accumulation of the resulting construct in some, but not all, tissues. For example, TAMRA-Tat preferably accumulated in the intestine, whereas TAMRA-Tat-NR2B9c and TAMRA-Tat-*N*-dimer preferably accumulated in the liver and kidney, respectively.

We detected 0.4% ± 0.1% of injected dose/g of TAMRA-Tat in mouse brain homogenate, which is in line with a previous study [23] reporting 0.1% of injected dose/g of ^111^In-DOTA-labelled Tat (GRKKRRQRRRPPQ) following 1-h circulation in mice. Moreover, less TAMRA-Tat-NR2B9c and TAMRA-Tat-*N-*dimer appeared to accumulate in the brain compared to TAMRA-Tat. This observation is in agreement with the observed BBB permeation by the Tat-conjugated peptides in live mice, supporting our earlier notion that cargo conjugation to Tat impacts its resulting brain delivery.

The transferrin receptor is highly expressed at the brain capillaries compared to most other tissues [47], offering, in principle, the opportunity to target the BBB over other tissues for purposes of delivering drug to the brain. Therefore, transferrin receptor binding antibodies [48,49] and peptides [50,51,52] have been developed as potential drug delivery vectors in attempts to facilitate receptor-mediated transcytosis across the BBB. Yu et al. reported the accumulation of different transferrin receptor binding antibodies in mice brain homogenates, ranging from 0.1% to 0.6% injected dose/g [49]. Another study reported up to 0.4% injected dose/g of transferrin receptor antibody-coated gold nanoparticles in mouse brain homogenates [53]. Despite their theoretical affinity for the BBB over other tissues, the transferrin antibody-coated gold nanoparticles accumulated in off-target tissues to a similar extent as the Tat-conjugated peptides in the present study. Thus, TAMRA-Tat, TAMRA-Tat-NR2B9c, and TAMRA-Tat*-N*-dimer accumulate in the brain to a similar extent as antibodies designed to target the BBB. Notably, we compared relatively small peptides with large antibodies or nanoparticles and cannot make a one-to-one comparison, but the lack of approved biologics with documented ability to permeate the BBB makes it difficult to identify relevant benchmark compounds.

## 5. Conclusions

We demonstrated that the nature of a peptide-based cargo (NR2B9c or *N-*dimer) when conjugated to Tat affects the ability of the resulting construct to permeate the BBB in vitro and in vivo. We also demonstrated accumulation in the mouse brain to a similar extent as other relevant macromolecular therapeutics. However, the earlier observed difference between the relative target affinity of Tat-*N-*dimer in vitro compared to Tat-NR2B9c and the therapeutic effect in vivo cannot be explained solely by differences in their potential to permeate the BBB. We demonstrated that the chemical stability of the resulting Tat-conjugates and their ability to adhere to the plasma membrane of BCECs are cargo-dependent. Both the chemical stability and plasma membrane adherence are likely to impact the BBB permeating potential of Tat-conjugates. Furthermore, the chemical stability must be considered before drawing conclusions based on detection via a label, such as TAMRA in the present study. The mechanism by which Tat facilitates BBB permeation by NR2B9c and *N*-dimer was studied. A fraction of the peptides segregated to lysosomes upon vesicular uptake, limiting the total amount potentially destined for transcytosis across the BBB. Despite a significant decrease in the measured TEER, only a minor increase in FITC-dextran transport was observed in the presence of TAMRA-Tat and TAMRA-Tat-NR2B9c. This finding raises the question of paracellular permeation as the major route of BBB permeation. We observed a correlation between in vitro BCEC uptake and in vivo brain uptake, but further studies are needed to determine the degree to which transcellular and/or paracellular transport mechanisms contribute to the net BBB permeation by TAMRA-Tat, TAMRA-Tat-NR2B9c, and TAMRA-Tat-*N*-dimer. Our findings imply that Tat conjugation to a peptide-based therapeutic is not a one-size-fits-all strategy for brain drug delivery. Therefore, a detailed analysis in terms of stability, as well as BBB uptake and permeation by each Tat-cargo construct, must be conducted early in the discovery phase.

## Supplementary Materials

The following are available online at https://www.mdpi.com/1999-4923/12/7/661/s1. Figure S1. (A–D) Degradation profiles illustrated as chromatograms obtained at 498 nm (TAMRA detection) from UPLC analysis of TAMRA-NR2B9c, TAMRA-Tat, TAMRA-Tat-NR2B9c, and TAMRA-Tat-*N*-dimer before and after 4 h incubation in 37 °C media supplemented with 10% FBS. (E) UPLC chromatogram obtained at 498 nm of TAMRA as single entity. Figure S2. Remaining peptide after 4 h incubation in 37 °C PBS. Data are presented ± SD (N = 2). Figure S3. 100 µM TAMRA-Tat, TAMRA-Tat-NR2B9c, or TAMRA-Tat-*N*-dimer was applied to the *in vitro* bovine blood-brain barrier model for 15 min. Following cell fixation, peptide uptake into the endothelial cells and the astrocytes was inspected via TAMRA-fluorescence using confocal microscopy with co-staining of ZO-1 and β-actin, respectively. Z-stacks, scale bar: 10 µm. Figure S4. 10 µM or 100 µM TAMRA was applied to the *in vitro* bovine blood-brain barrier model for 3 h. Following cell fixation, potential TAMRA uptake into the endothelial cells and the astrocytes was inspected using confocal microscopy with co-staining of ZO-1 and β-actin, respectively. Z-stacks, scale bar: 10 µm. Figure S5. 100 µM TAMRA-NR2B9c, TAMRA-Tat, TAMRA-Tat-NR2B9c, or TAMRA-Tat-*N*-dimer was applied to an *in vitro* blood-brain barrier model composed of primary mouse endothelial cells in co-culture with primary rat astrocytes for 3 h. (A) Total peptide being transported across the barrier was quantified. (B) After the permeation study, the cells were washed with HBSS prior quantification of peptide accumulating in the cell fraction. Data are presented as mean ± SEM (N = 3, *n* = 3) Levels of significance are *: p < 0.05 and **: p < 0.01 when compared to TAMRA-NR2B9c (one-way ANOVA with Dunnett’s multiple comparisons test). Figure S6. Primary mouse cortical neurons were incubated with 1 µM TAMRA-Tat, TAMRA-Tat-NR2B9c, or TAMRA-Tat-*N*-dimer for 1 h at 37 °C. Following cell fixation, peptide uptake was inspected by confocal microscopy via TAMRA fluorescence with co-staining of β-actin. Single xy section, scale bar: 10 µm. Figure S7. Physiological parameters after 1 h peptide circulation during two-photon imaging of peptide blood-brain barrier permeation in live mice: (A) Electrocorticographic activity (ECoG), (B) exhaled CO_2_, and (C) mean arterial blood pressure (MABP). Data are presented as mean ± SEM (N = 5). Levels of significance are * p < 0.05; ** p < 0.01 (two-tailed unpaired *t*-test).
