# Supplementary material for: Conjugation of Therapeutic PSD-95 Inhibitors to the Cell-Penetrating Peptide Tat Affects Blood–Brain Barrier Adherence, Uptake, and Permeation
Source: Pharmaceutics. 2020 Jul 14;12(7):661. doi: 10.3390/pharmaceutics12070661 (PMC7408072; doi:10.3390/pharmaceutics12070661)
Supplement: Supplementary file 1 [file pharmaceutics-12-00661-s001.pdf]

pharmaceutics

MDPI

Supplementary Materials: Conjugation of Therapeutic PSD-95 Inhibitors to the Cell-Penetrating Peptide Tat Affects Blood-Brain Barrier Adherence, Uptake, and Permeation

Mie Kristensen, Krzysztof Kucharz, Eduardo Felipe Alves Fernandes, Kristian Strømgaard, Morten Schallburg Nielsen, Hans Christian Cederberg Helms, Anders Bach, Malte Ulrikkaholm Tofte-Hansen, Blanca Irene Aldana Garcia, Martin Lauritzen and Birger Brodin

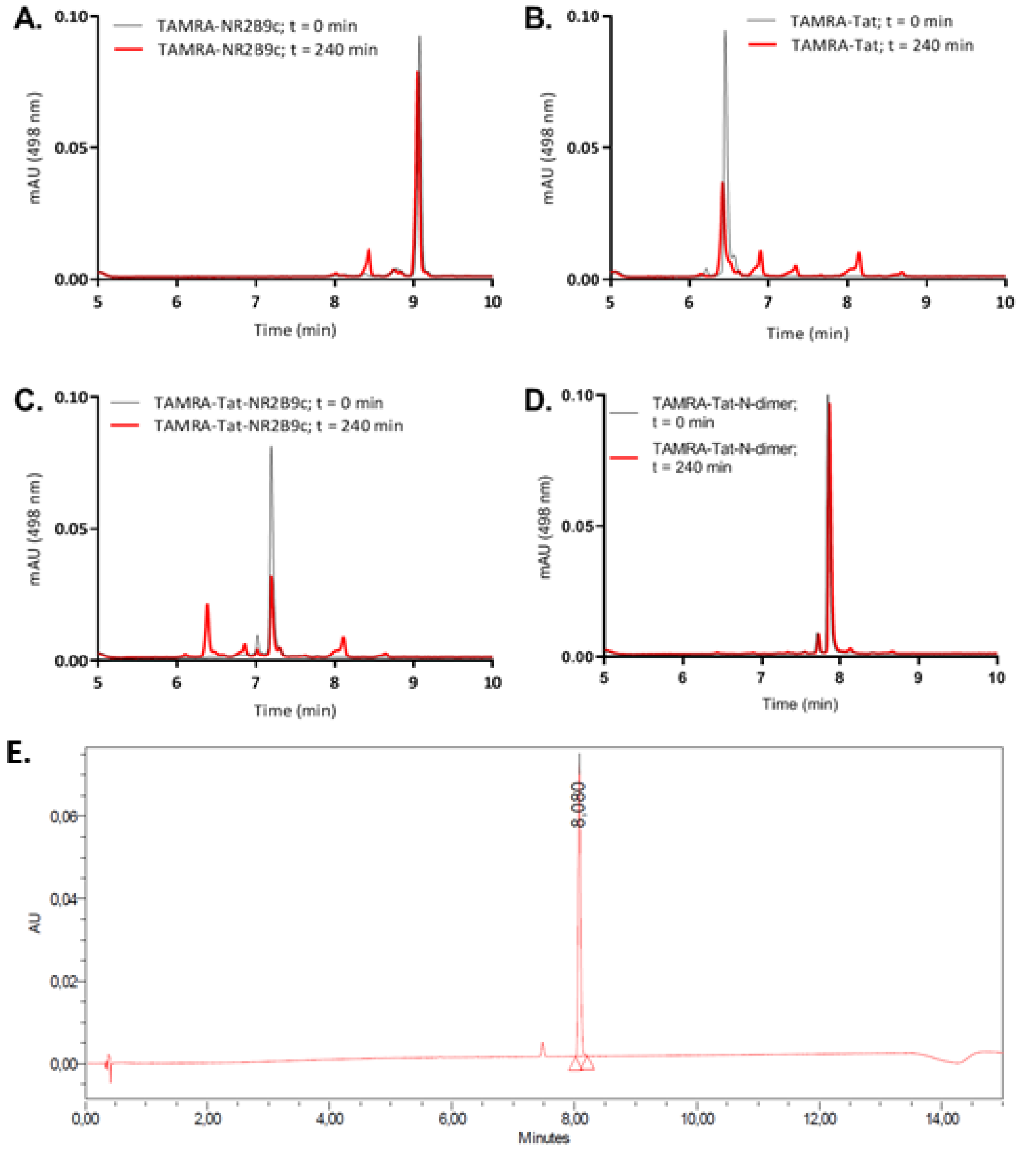

Figure S1. (**A–D**) Degradation profiles illustrated as chromatograms obtained at 498 nm (TAMRA detection) from UPLC analysis of TAMRA-NR2B9c, TAMRA-Tat, TAMRA-Tat-NR2B9c, and TAMRA-Tat-*N*-dimer before and after 4 h incubation in 37 °C media supplemented with 10% FBS. (**E**) UPLC chromatogram obtained at 498 nm of TAMRA as single entity.

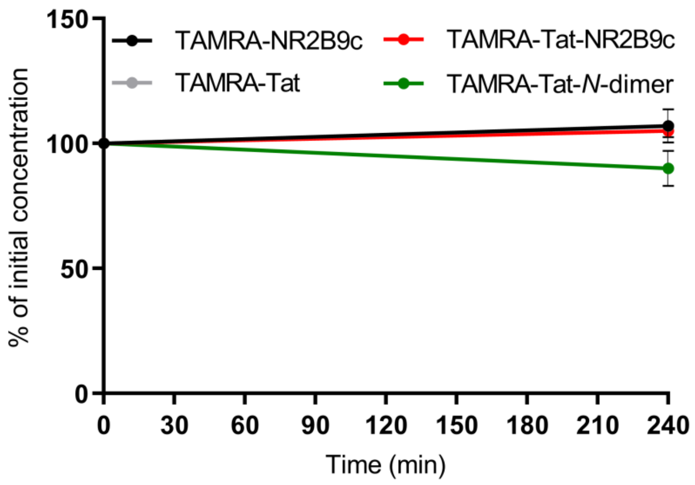

Figure S2. Remaining peptide after 4 h incubation in 37 °C PBS. Data are presented ± SD (N = 2).

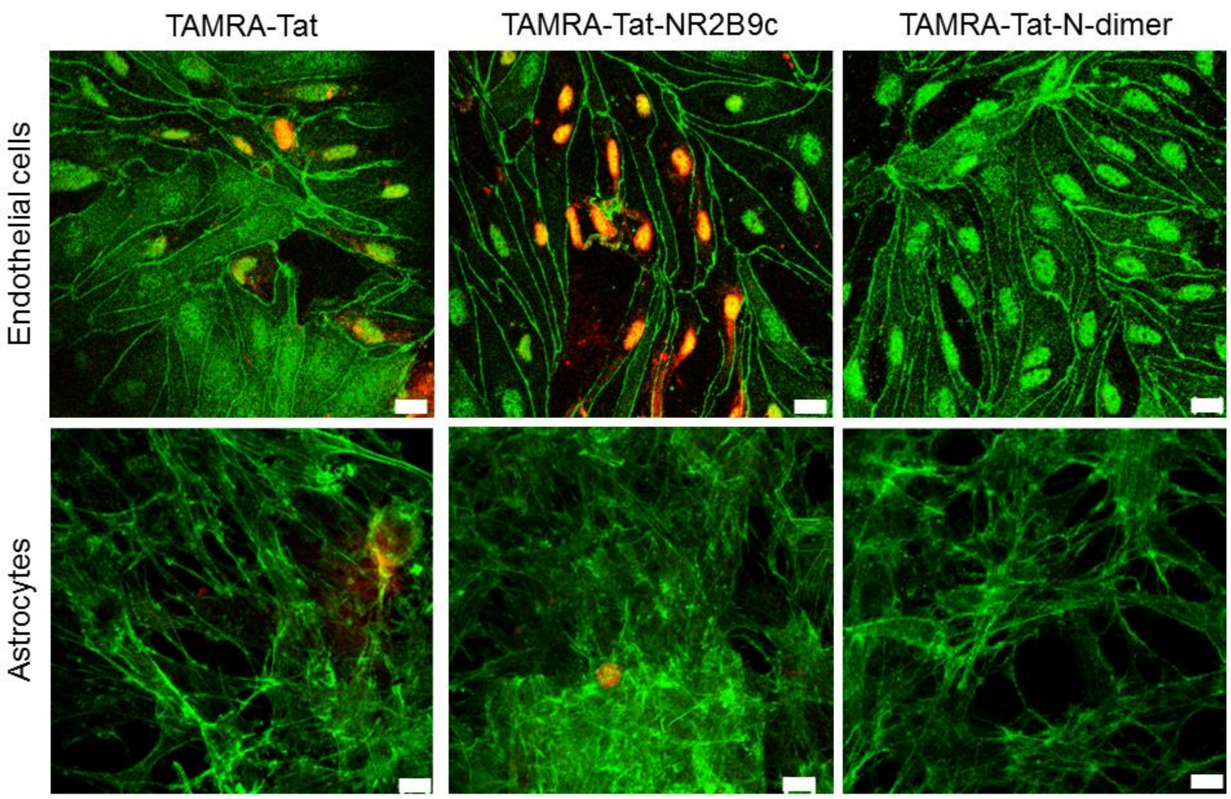

Figure S3. 100 µM TAMRA-Tat, TAMRA-Tat-NR2B9c, or TAMRA-Tat-*N*-dimer was applied to the *in vitro* bovine blood-brain barrier model for 15 min. Following cell fixation, peptide uptake into the endothelial cells and the astrocytes was inspected via TAMRA-fluorescence using confocal microscopy with co-staining of ZO-1 and β-actin, respectively. Z-stacks, scale bar: 10 µm.

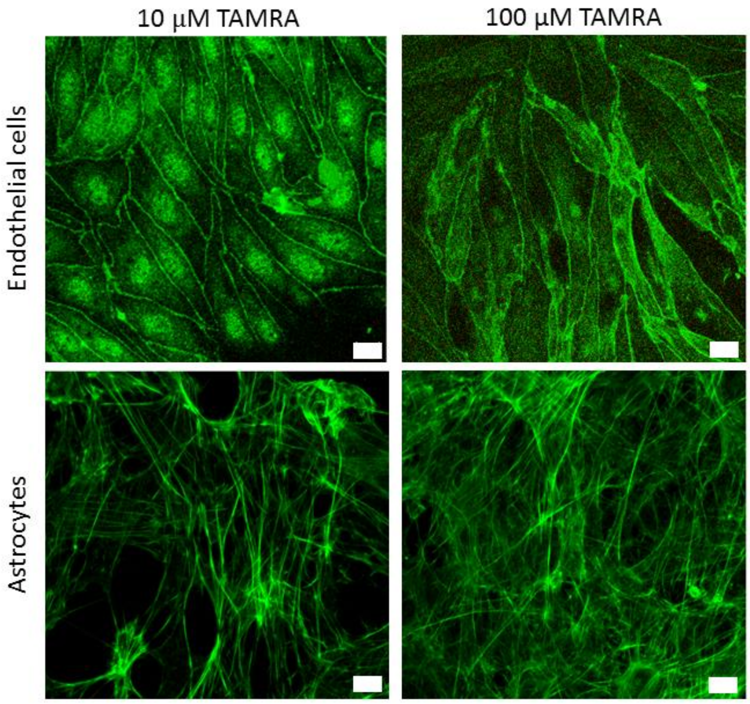

Figure S4. 10 µM or 100 µM TAMRA was applied to the *in vitro* bovine blood-brain barrier model for 3 h. Following cell fixation, potential TAMRA uptake into the endothelial cells and the astrocytes was inspected using confocal microscopy with co-staining of ZO-1 and β-actin, respectively. Z-stacks, scale bar: 10 µm.

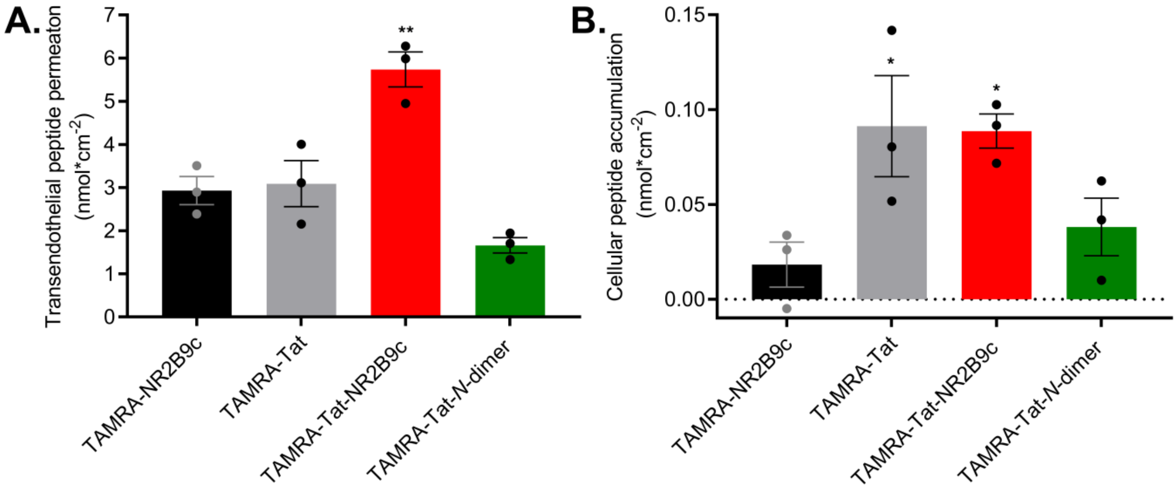

Figure S5. 100 µM TAMRA-NR2B9c, TAMRA-Tat, TAMRA-Tat-NR2B9c, or TAMRA-Tat-*N*-dimer was applied to an *in vitro* blood-brain barrier model composed of primary mouse endothelial cells in co-culture with primary rat astrocytes for 3 h. (**A**) Total peptide being transported across the barrier was quantified. (**B**) After the permeation study, the cells were washed with HBSS prior quantification of peptide accumulating in the cell fraction. Data are presented as mean ± SEM (N = 3, *n* = 3) Levels of significance are *: $p < 0.05$ and **: $p < 0.01$ when compared to TAMRA-NR2B9c (one-way ANOVA with Dunnett's multiple comparisons test).

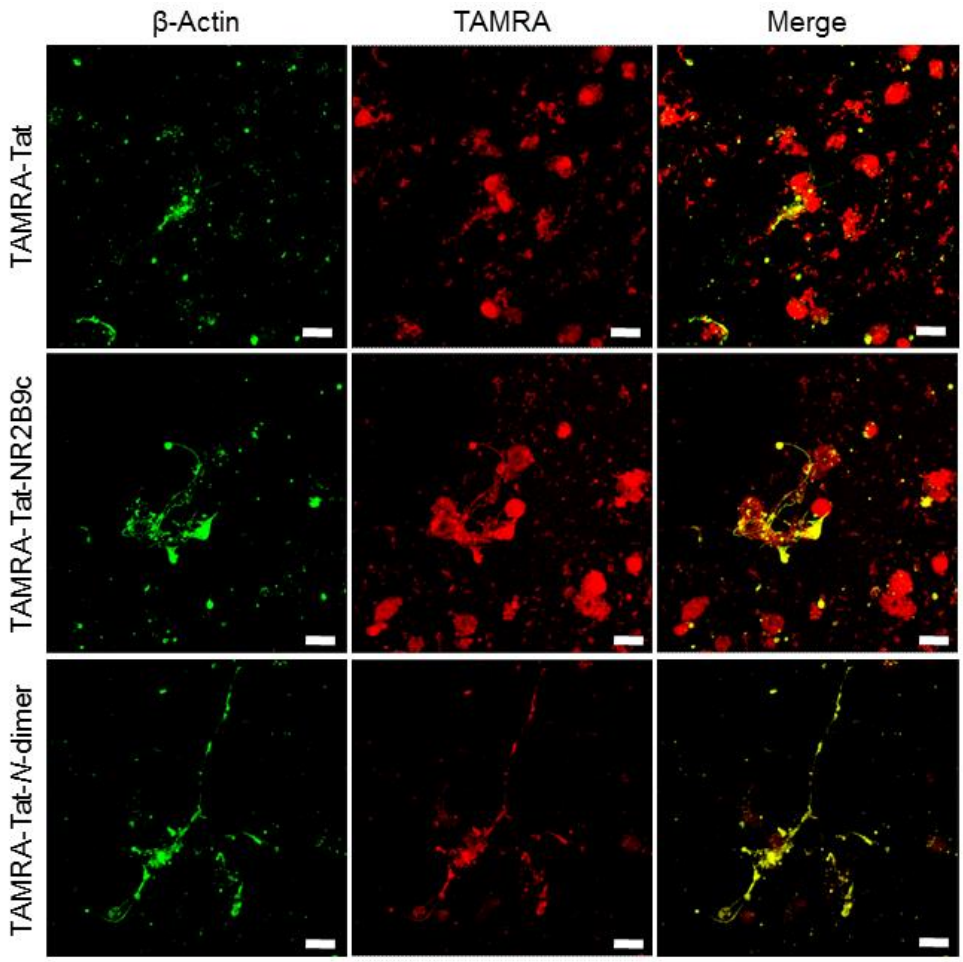

Figure S6. Primary mouse cortical neurons were incubated with 1 μM TAMRA-Tat, TAMRA-Tat-NR2B9c, or TAMRA-Tat-*N*-dimer for 1 h at 37 °C. Following cell fixation, peptide uptake was inspected by confocal microscopy via TAMRA fluorescence with co-staining of β-actin. Single xy section, scale bar: 10 μm.

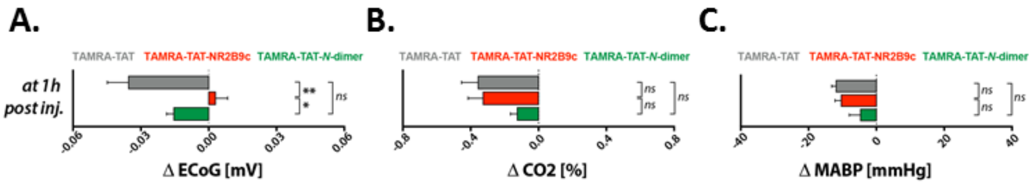

Figure S7. Physiological parameters after 1 h peptide circulation during two-photon imaging of peptide blood-brain barrier permeation in live mice: (**A**) Electrocorticographic activity (ECoG), (**B**) exhaled CO_2, and (**C**) mean arterial blood pressure (MABP). Data are presented as mean ± SEM (N = 5). Levels of significance are * $p < 0.05$; ** $p < 0.01$ (two-tailed unpaired *t*-test).
